# Using bioelectrical impedance analysis for modeling regression and predicting body fat accumulation in dogs in East Thailand

**DOI:** 10.14202/vetworld.2022.1566-1574

**Published:** 2022-06-28

**Authors:** Peera Arreesrisom, Thanmaporn Phichitrasilp, Nattakarn Naimon, Supochana Charoensin, Worawut Rerkamnuaychoke, Jumrueang Panpiansil, Thitichai Jarudecha

**Affiliations:** 1 Department of Veterinary Technology, Faculty of Veterinary Technology, Kasetsart University, 50 Ngam Wong Wan Rd, Ladyaow, Chatuchak, Bangkok, Thailand; 2 Faculty of Veterinary Medicine, Rajamangala University of Technology Tawan-ok, 43 Moo 6 Bangpra, Sriracha, Chonburi, Thailand; 3 Mueangchonsattawarak Co. Ltd., 843/2, 843/70, Soi Taladmai Bankhod, Mueang Chonburi, Thailand

**Keywords:** bioelectric impedance analysis, body fat, dog, Thailand

## Abstract

**Background and Aim:**

Obesity in dogs leads to several health problems, such as premature death, and contributes to other diseases. Recently, body fat percentage has been considered to represent the body condition of dogs, and bioelectrical impedance analysis (BIA) is the most effective method for accurately measuring body fat in dogs. In Thailand, information on the body condition of dogs is limited, and there is no standard body fat level for Thai or mongrel dogs. This study was designed to evaluate and analyze the body fat percentage in dogs through BIA using a handheld instrument. The results of this study can help enhance the quality of life and health of dogs and aid in setting a standard body fat level for Thai or mongrel dogs.

**Materials and Methods:**

The body fat percentage of 340 Thai and mongrel dogs in East Thailand was measured in the standing position, and the body condition score (BCS) (range, 1–5), sex, sterilization status, age, type of diet, and lifestyle were recorded. A linear regression model was developed to compare the variables and the predicted body fat percentage, and multiple linear regressions were used to analyze the factors for body fat increment.

**Results:**

The linear regression model used to estimate the percentage of body fat (y) for each BCS (x) was y = 0.84 + 8.36x (R^2^ = 0.7219; p < 0.0001); the average body fat percentage was 27.52% for all studied dogs; specifically, 24.83% for the Thai Bangkaew, 26.42% for the Thai Ridgeback, and 27.65% for mongrels. The median body fat percentage was significantly higher in female than in male dogs. We found that as age increases, body fat percentage also increases; this increasing trend begins at the age of 5 years. However, increasing the level of activity and decreasing meal frequency leads to an increase in body fat percentage in neutered male dogs.

**Conclusion:**

The average body fat percentage of dogs in East Thailand is 27.52% and this value is expected to increase when these dogs reach the age of 5 years. BIA is a valid and effective measurement tool for detecting the body fat percentage in dogs.

## Introduction

Humans are considered overweight and obese when the body accumulates an excessive amount of fat. Overweight is defined as a body mass index (BMI) of ≥ 25 kg/m^2^, and obesity is defined as a BMI of ≥ 30 kg/m^2^. It is estimated that 38% of the world’s adult population will be overweight and 20% will be obese by 2030 [1]. Previously, overweight or obesity in dogs has been determined on the basis of body weight and body condition score (BCS; a 9-point scale), both of which are common methods for assessing the nutritional status or body weight of small animals. Dogs are generally classified as overweight when they weigh 10%–20% over their ideal body weight and are considered obese if their weights are >20% [2].

BCS is measured using visual and palpation techniques; dogs are categorized as overweight and obese when they have a BCS of 4 and 5 levels, respectively [3]. Even though BCS is a recognized parameter for assessing the nutritional status of small animals, it is subjective because the evaluation uses visual and palpation techniques. Therefore, several tools have been developed and used to measure the body fat percentage in humans and animals that help enable the precise measurement of fat levels in less time and at a low cost. Dual-energy X-ray absorptiometry (DEXA) and BCS [4, 5] are additional methods for estimating body fat in dogs; however, DEXA is difficult to perform in clinical practice and involves exposing patients to radiation. At present, bioelectrical impedance analysis (BIA) is probably the best tool for measuring animal body fat. This method involves introducing a weak electrical current and measuring the resistance. BIA can be used regardless of the breed or body size of the animal and is particularly useful in monitoring dog obesity and health [6]. Both overweight and obesity in dogs are complex disorders [7, 8] and can cause respiratory problems [9]. A recent study determined that overweight and obesity can affect the serum concentration of symmetric dimethyl arginine [10]. Dog obesity causes several health problems and contributes to other disorders, such as musculoskeletal problems, respiratory distress, heart disease, and diabetes mellitus [11–16]. The most common cause of obesity is excessive energy intake over energy expenditure. In addition, several factors that lead to dogs becoming overweight have been identified, including breed, genetic background, neuter status, orthopedic diseases, type of diet, changes in lifestyle introduced by the owner, and the level of physical activity [2, 17–20]. It is expected that up to 40% of dogs in developed countries are overweight, thus indicating the widespread nature of the problem [21]. At present, it is estimated that > 35% of dogs are obese worldwide [22]. Dog obesity is observed to occur in middle-aged animals aged 5–10 years [2]. Obesity could shorten a dog’s life and increase the risk of several diseases. Overweight dogs have shorter life spans than lean dogs, usually by up to 6–12 months. However, a comprehensive lifetime study on Labrador Retrievers found that being moderately overweight could reduce a dog’s life expectancy by nearly 2 years compared with leaner animals [23]. For companion animal welfare, research on weight control and factors of dog obesity is ongoing [24–27].

According to the Bureau of Disease Control and Veterinary Services, there were approximately 7.3 million dogs in Thailand in 2016. Being overweight or obese threatens a dog’s health; therefore, information on dogs’ body fat is important to enable us to manage overweight or obese dogs in Thailand. This study was designed to analyze the body fat percentage in dogs and design a prediction model using a handheld BIA device (Kao Healthlab IBF-D02, Tokyo, Japan).

## Materials and Methods

### Ethical approval

Animal ethics approval was obtained from the Animal Ethics Committee of Kasetsart University (Approval no. ACKU60-VTN-001, and ACKU60-VTN-002), Bangkok, Thailand.

### Study period and location

The study was conducted from August 2015 to January 2018. The samples were collected from eastern region of Thailand. The samples and data were analyzed at Faculty of Veterinary Technology, Kasetsart University.

### Animals and study area

The criteria for sampling data were Thai or mongrel dogs, at least 1 year of age, good health, and did not receive any medications other than a heartworm or flea prevention. A sample of 340 dogs were collected from veterinary clin­ics in the eastern region of Thailand. All dogs were confirmed healthy by veterinarians.

### Experimental design

BCS (ranging from 1 to 5), sex, sterilization status, age, type of food, and lifestyle of all dogs in the study were recorded. The dogs were divided into four groups according to sex (i.e., intact male, neutered male, intact female, and spayed female groups); six groups according to age (i.e., 1–2, 3–4, 5–6 years old, 7–8, 9–10, and over 11); six groups according to food type (i.e., dry diet, cooked by owner, dry diet with dog food, dry diet with cooked by owner, canned dog food, and cooked by owner and canned food); four groups according to the frequency of meals per day (i.e., 1 time/day, 2 times/day, 3 times/day, and > 3 times/day); two groups according to the presence or absence of snacks; and three groups according to the frequency of letting activity outside the house area per day (i.e., never, 1, and > 1 time/day).

Body fat percentage was measured using IBF-D02 (Kao Corporation, Tokyo, Japan). This device is mainly used for detecting the body fat percentage in dogs. A study conducted by Stone *et al*. [5] used this device for detecting the body fat percentage in dogs. Some animal hospitals have used this device as a tool for managing canine diets. Therefore, the reliability of this device is high, and it can accurately detect body fat percentage in dogs. The BIA device was used with all dogs held in the standing position and all four electrodes making direct contact with the skin. The device functions by sending an electric pulse from electrode 1 to electrode 4, whereas electrode 2 sends an electric pulse to electrode 3. Subsequently, 70% alcohol was applied from the dorsal lumbar region to the last rib and 2 cm of the midline of the thoracic vertebrae of the dogs; the hair overlying the epaxial musculature was parted using a comb to expose the underlying skin. Three consecutive readings were obtained for each dog for approximately 60 s.

Five-point BCS was calculated based on the amount of fat covering the rib area assessed through visual inspection and palpation. The 5-point scores were as follows: 1 = very thin, 2 = underweight, 3 = ideal, 4 = overweight, and 5 = obese.

### Statistical analysis

All statistical analyses were performed using STATA (version 15.1, Stata Corporation, Texas, USA). Body fat percentage values were tested for normal distribution using the Shapiro–Wilk test, and normally distributed data were expressed as means ± standard deviations, whereas non-normally distributed data were presented as medians and ranges. The significance level was tested using the two-sample Wilcoxon rank-sum (Mann–Whitney) test for analysis between sex types and the Kruskal–Wallis test for comparisons of the median body fat percentage between females, males, spayed females, and neutered males. For normally distributed data, the body fat percentage between the age groups of female dogs was analyzed using a one-way analysis of variance, and for the non-normally distributed data, the body fat percentage between the age groups of male dogs and both sexes were analyzed using the Kruskal–Wallis test with the statistical level set at p ≤ 0.05. A linear regression model was used to predict the body fat percentage. Multiple linear regressions were performed using the body fat percentages compared within the aforementioned variables (i.e., age range, type of food, frequency of meals per day, presence or absence of snacks, and frequency of letting activity outside the house area per day). In the multivariate model, variables with a univariate p ≤ 0.10 by simple linear regression were added. The normality, homoscedasticity, correlation, and multicollinearity of the variables were checked for multiple linear regressions. Quantile regression analysis was used for non-normal distribution; multiple linear regression with robust variance estimates was used to detect heteroscedasticity in the multivariate model.

## Results

**Figure-1:**
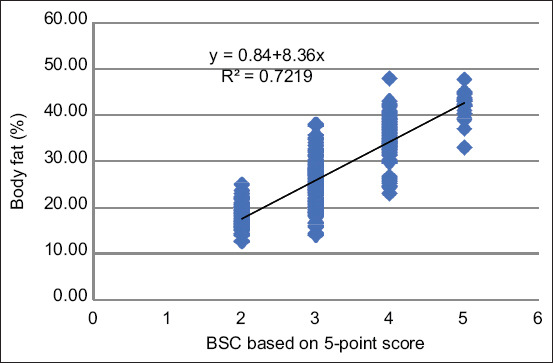
Linear regression model (y = 0.84 + 8.3606x) predicting percentage of body fat using body condition score (BSC; R^2^ = 0.7219). Each additional 1 point from BSC 2 to 5 reflects an 8.36% increase in body fat.

A linear regression model (y = 0.84 + 8.36x) was constructed to measure the body fat percentage using mean BCS (R^2^ = 0.7219; 72.19%). According to this model, each additional increase in the BCS by 1 between scores of 2 and 5 reflected an increase in the body fat percentage by approximately 8.36% using the BIA device (curve slope, 8.36; 95% confidence interval = 7.81–8.92; p < 0.0001). Using the linear regression model, the estimated body fat percentages for each BCS class were 17.56%, 25.92%, 34.28%, and 42.64%, respectively (Figure-1). According to the linear regression model, the body fat percentages could be classified into five groups: <17.56%, very thin group; 17.56–25.91%, underweight group; 25.92–34.27%, ideal group; 34.28–42.64%, overweight group; and >42.64%, obese group. The body fat percentages of the dogs in the very thin, underweight, ideal, overweight, and obese groups were 12.65%, 34.41%, 29.12%, 20.59%, and 3.23%, respectively. The underweight group had the highest number (34.41%) of dogs. Neutered male dogs had a higher body fat percentage than intact male dogs, except for those in the underweight group. Spayed female dogs also had a higher body fat percentage than intact female dogs, except for those in the very thin group. The combination of overweight and obese dogs accounted for 23.82% of all dogs in the study, and their classification according to sex was as follows: 17.64% in males, 27.63% in neutered males, 17.33% in intact females, and 30.77% in spayed females (Table-1). The average body fat percentage of all dogs in this study was 27.52%, and the average body fat percentages by breed were as follows: 24.83% in the Thai Bangkaew, 26.42% in the Thai Ridgeback, and 27.65% in mongrels (Table-2).

**Table 1:** Percentage of body fat classified by sex of dog.

Sex	Body fat (%)				

	<17.56% (Very thin)	17.56–25.92% (Underweight)	25.92–34.28% (Ideal)	34.28–42.64 (Overweight)	>42.64 (Obesity)
Intact male	13 (15.29)	38 (44.71)	19 (22.35)	13 (15.29)	2 (2.35)
Neutered male	16 (21.05)	20 (26.32)	19 (25.00)	18 (23.68)	3 (3.95)
Intact female	8 (10.67)	25 (33.33)	29 (38.67)	11 (14.67)	2 (2.66)
Spayed female	6 (5.77)	34 (32.69)	32 (30.77)	28 (26.92)	4 (3.85)
Total	43 (12.65)	117 (34.41)	99 (29.12)	70 (20.59)	11 (3.23)

**Table 2:** The average percentage of body fat by dog breed.

Breed	Thai Ridgeback (n = 4)	Thai Bangkaew (n = 7)	Mongrel (n = 329)	Total (n = 340)
Body fat (%)	26.42	22.48	27.65	27.52

We compared the body fat percentage between sexes and found that the median body fat percentage was significantly different between female (28%) and male (24.67%) dogs (Table-3). The average body fat percentages in intact female, spayed female, intact male, and neutered male dogs were 27.67%, 28.33%, 24%, and 26.5%, respectively. However, the median body fat percentage in female dogs was significantly higher than that in male dogs, and the median body fat percentage in spayed female dogs was significantly higher than that in male dogs; however, no significant difference in the median body fat percentage was observed between spayed female dogs and neutered male dogs (Table-4).

**Table 3:** Body fat versus sex of dogs.

Parameter	Body fat (%)			95% CI difference	p-value

	n	Median	Range		
Female (x̅ = 28.71)	179	28	14–48	1–4.67	0.002*
Male (x̅ = 26.21)	161	24.67	12.67–44.67		

* Significant difference, comparison of medians between groups performed using two-sample Wilcoxon rank-sum (Mann–Whitney) test and P *≤* 0.05 considered statistically significant.

**Table 4:** Comparison of body fat percentage of dogs.

Parameter	Body fat (%)			95% CI	p-value

	n	Median	Range		
Female					
Intact (x̅ = 27.52)	75	27.67^a^	14.33–43.33	25.16–30	0.004*
Spayed (x̅ = 29.56)	104	28.33^b^	14–48	26–32.17	
Male					
Intact (x̅ = 25.31)	87	24^a,b^	12.67–44.67	22–25.42	
Neutered (x̅ = 27.27)	74	26.5	12.67–44.67	21.85–30.25	

* Significant difference, comparison of medians between groups, performed using Kruskal–Wallis test and

a,b comparison of medians between groups performed using two-sample Wilcoxon rank-sum (Mann–Whitney) test. *P ≤*0.05 considered statistically significant (

a p = 0.0356,

b p = 0.0003).

The body fat percentage of female dogs aged ≥5 years was significantly higher than that of those aged 1–2 years, regardless of the food type and frequency of activity outside the house (Tables-5 and 6); however, this value was not significantly different for those on a dry diet and those eating other foods, after adjusting for other variables (Table-6). The body fat percentage of spayed female dogs fed ≥1 time/day did not significantly differ from that of those in the never letting group (Table-6). Moreover, the body fat percentage of intact male dogs aged ≥5 years was significantly higher than that of those aged 1–2 years, after adjusting for other variables. The body fat percentages of neutered male dogs aged 7–8 years and >11 years were significantly higher than those of neutered male dogs aged 1–2 years, after adjusting for other variables (Tables-5 and 6). Moreover, the body fat percentage of neutered male dogs that had activity outside the house >1 time/day or had snacks were significantly higher than that of those who never had activity outside the house or did not have snacks; however, the body fat percentage of neutered male dogs that had >1 meal/day was significantly lower than that in those who had one meal/day after adjusting for other variables (Tables-5 and 6).

**Table 5:** Body fat (%) of male and female dogs in all variables.

Variable	Female						Male					
	
	Intact			Spayed			Intact			Neutered		
			
	n	Mean (SD)	Median (IQR)	n	Mean (SD)	Median (IQR)	n	Mean (SD)	Median (IQR)	n	Mean (SD)	Median (IQR)
Age range												
1–2 years	38	25.06 (6.43)	24.33 (9.67)	48	25.85 (6.60)	25.17 (9.66)	27	22.27 (5.31)	21.33 (7.33)	31	22.78 (7.10)	20.67 (10.00)
3–4 years	15	26.31 (6.34)	27.33 (13.67)	15	27.18 (6.84)	26.00 (4.66)	23	27.04 (10.34)	23.67 (20.33)	14	20.74 (6.16)	17.67 (10.33)
5–6 years	6	31.17 (7.24)	33.67 (4.33)	10	32.73 (7.46)	34.67 (13.67)	12	24.11 (8.02)	23.17 (14.50)	14	33.12 (7.48)	34.50 (12.34)
7–8 years	9	34.78 (6.57)	33.67 (5.33)	14	35.05 (7.34)	36.67 (6.33)	8	30.50 (5.27)	30.50 (8.17)	5	38.27 (5.57)	41.00 (1.00)
9–10 years	2	34.67 (1.89)	34.67 (2.67)	7	34.43 (9.10)	34.00 (17.67)	6	27.44 (5.01)	25.83 (6.33)	6	30.83 (7.79)	32.33 (11.00)
≥ 11 years	5	29.60 (4.05)	28.33 (6.33)	10	36.67 (7.30)	38.50 (7.67)	9	24.89 (5.43)	24.00 (5.67)	6	39.61 (5.11)	40.67 (4.67)
Food type												
Dry diet	12	25.22 (5.76)	24.50 (8.50)	14	28.29 (9.12)	29.00 (17.00)	8	23.00 (5.88)	21.00 (6.50)	16	23.21 (9.26)	19.33 (13.00)
Cooked by owner	18	26.32 (7.44)	25.00 (12.34)	32	31.58 (8.04)	32.83 (14.00)	32	22.83 (6.77)	21.33 (9.17)	24	29.07 (9.54)	26.83 (19.00)
Dry diet with food dog	9	22.52 (6.62)	20.33 (7.66)	5	33.67 (6.46)	35.00 (5.34)	1	24.67 (N/A)	24.67 (N/A)	0	N/A	N/A
Dry diet with cooked by owner	34	30.60 (6.24)	32.00 (7.66)	51	27.90 (7.55)	27.00 (11.33)	42	27.57 (8.27)	27.50 (12.00)	34	28.08 (8.98)	27.83 (15.33)
Canned dog food	1	28.33 (N/A)	28.33 (N/A)	0	N/A	N/A	0	N/A	N/A	0	N/A	N/A
Cooked by owner and canned food	0	N/A	N/A	1	48.00 (N/A)	48.00 (N/A)	1	23.00 (N/A)	23.00 (N/A)	0	N/A	N/A
Meal/day												
1 time/day	19	23.81 (6.71)	21.00 (10.00)	22	29.38 (9.32)	26.50 (15.67)	9	21.78 (6.57)	19.33 (9.33)	16	33.81 (10.07)	38.83 (17.00)
2 times/day	37	28.27 (6.43)	28.33 (10.67)	69	29.51 (7.81)	28.33 (11.00)	53	24.92 (7.80)	23.00 (10.67)	47	25.90 (8.91)	25.67 (13.00)
3 times/day	8	27.38 (8.30)	29.67 (13.33)	6	26.06 (7.99)	26.50 (11.00)	7	30.86 (7.91)	32.67 (11.33)	4	27.50 (5.78)	27.33 (8.00)
> 3 times/day	8	32.17 (7.76)	32.83 (5.50)	5	32.20 (8.32)	34.67 (6.67)	15	26.64 (6.07)	24.33 (7.67)	7	23.62 (7.26)	18.67 (14.00)
Snacks												
No	46	27.17 (6.74)	28.00 (11.67)	94	28.92 (8.02)	28.00 (11.67)	65	24.93 (7.99)	23.67 (10.67)	37	31.23 (8.54)	31.00 (14.00)
Yes	8	29.71 (9.64)	32.17 (16.33)	4	29.75 (5.42)	29.84 (8.84)	12	26.22 (7.08)	25.33 (12.67)	6	37.72 (6.04)	37.67 (9.00)
Letting activity outside house												
Never	58	28.63 (6.73)	28.67 (10.67)	90	28.68 (7.83)	27.67 (11.67)	67	24.43 (7.71)	23.00 (9.33)	69	26.48 (9.30)	26.00 (16.00)
1 time/day	7	24.52 (9.20)	21.67 (18.67)	7	39.14 (5.27)	37.67 (7.66)	4	26.42 (5.65)	27.17 (10.00)	4	33.83 (7.46)	34.83 (12.00)
> 1 time/day	3	27.89 (7.15)	31.33 (13.00)	7	31.33 (8.69)	33.33 (14.33)	13	29.10 (7.41)	28.67 (9.67)	3	37.33 (2.85)	37.00 (5.66)

**Table 6:** Estimation of body fat (%) using the multivariable regression analysis.

Variable	Female				Variable	Male			
	
	Intact^a^		Spayed^b^			Intact^a^		Neutered^c^	
			
	B (SE)	p-value	B (SE)	p-value		B (SE)	p-value	B (SE)	p-value
Constant	21.667 (1.778)	<0.001	26.238 (1.894)	<0.001	Constant	24.000 (1.053)	<0.001	31.421 (3.005)	<0.001
Age range					Age range				
1-2 years	Reference		Reference		1-2 years	Reference		Reference	
3-4 years	0 (N/A)	1.000	0.451 (2.083)	0.829	3-4 years	1.333 (1.852)	0.472	0.839 (5.805)	0.886
5-6 years	6.667 (2.061)	0.001	7.102 (2.447)	0.005	5-6 years	7.000 (2.272)	0.002	5.130 (3.607)	0.166
7-8 years	10.333 (2.326)	<0.001	7.887 (2.180)	<0.001	7-8 years	9.333 (2.197)	<0.001	8.485 (2.487)	0.002
9-10 years	9.000 (2.899)	0.002	8.494 (2.822)	0.003	9-10 years	6.000 (2.959)	0.044	1.995 (4.198)	0.638
≥11 years	7.667 (2.481)	0.002	9.257 (2.813)	0.001	≥11 years	5.000 (2.445)	0.042	10.985 (2.950)	0.001
Food type					Letting activity outside house				
Dry diet	Reference		Reference		Never	Reference		Reference	
Owner cook	-0.667 (2.019)	0.742	1.536 (2.280)	0.502	1 time/day	4.333 (2.655)	0.104	2.476 (2.531)	0.336
Dry diet with food dog	-1.333 (3.499)	0.704	1.929 (3.785)	0.612	>1 time/day	2.333 (2.357)	0.323	6.423 (1.763)	0.001
Dry diet with owner cook	3.667 (1.895)	0.054	-1.781 (2.116)	0.402	Meal/day				
Canned dog food	-1.000 (10.148)	0.922	N/A	N/A	1 time/day			Reference	
Owner cook and canned food	-7.667 (10.232)	0.455	7.172 (7.871)	0.365	2 times/day			-5.479 (2.299)	0.024
Letting activity outside house					3 times/day			-11.164 (1.944)	<0.001
Never			Reference		>3 times/day			-11.780 (3.381)	0.002
1 time/day			5.334 (3.277)	0.107	Snacks				
>1 time/day			0.005 (2.795)	0.999	No			Reference	
					Yes			5.970 (2.674)	0.033

Statistical analysis by quantile regression (a), multiple linear regression (b) and multiple linear regression with robust variance estimates (c). Comparison of mean or median of body fat between variable and reference. p ≤ 0.05 were considered significant and N/A is not applicable.

## Discussion

Obesity is a risk factor for other diseases in dogs as well as humans. Analyzing the body fat percentage in dogs is mostly a complicated method. Previously, the most widely accepted and practical method for evaluating body condition was condition scoring through visual assessment and palpation, such as through BCS, morphometric measurements, and BCS using a 9-point scale [4]. This method was performed according to the descriptions and illustrations provided by the World Small Animal Veterinary Association. Recently, several devices have been developed to detect body fat. DEXA uses two X-ray beams at two different energy levels to estimate the bone mineral content and soft-tissue composition. DEXA can be used to estimate the body composition of dogs [28, 29]. However, this device is expensive and has limited practical application in veterinary practice. BIA is a reliable and accessible test for screening body composition in both humans and animals. BIA measures the resistance to an electrical signal through water that is found in muscle and fat. The more muscle a patient has, the more water the patient can hold; the greater the amount of water, the easier it is for the current to flow through it. As the amount of fat increases, the resistance to the current increases as well [30]. BIA is the most effective method for detecting body fat as it is accurate, time and cost-efficient, and easy to use. In this study, we used a handheld BIA device (Kao Healthlab IBF-D02) and the 5-point BCS system as tools to detect the body fat percentage in 340 dogs in Thailand. The results of this study can be used to measure and manage body fat levels in dogs and help develop a standard measurement tool for determining body fat levels in Thai or mongrel dogs.

The exact method through which body fat percentage can be used to categorize dogs as normal, overweight, or obese remains unclear; however, body fat percentage was significantly correlated with BCS [31]. Some studies have estimated that dogs with body fat percentages of 15–20%, 25–40%, and > 40% should be categorized as normal, overweight, and obese dogs, respectively [4, 28, 32, 33]. However, other studies have reported that the body fat percentage of normal dogs was 15–22% for male and neutered male dogs and 15–25% for female and spayed female dogs. Other studies have indicated that male and female dogs with body fat percentages exceeding 22% and 25%, respectively, should be considered overweight and that if their body fat percentage was > 36.60%, they should be classified as obese [5, 22].

In this study, we used a 5-point BCS system and constructed a linear regression model (y = 0.84 + 8.36x) to estimate the body fat percentage using a mean BCS score (R^2^ = 0.7219; 72.19%). Linear regression analysis revealed that a body fat percentage of < 17.56% should be classified as very thin, 17.56–25.91% as underweight, 25.92–34.28% as ideal weight, 34.28–42.63% as overweight, and > 42.63% as obese.

Dogs are considered obese when their body weight exceeds the optimum value [2]. The incidence of obesity is high in neutered or spayed dogs. Obesity in older dogs can be caused by several causes, such as a reduced metabolic rate that occurs with aging and the loss of sex hormones, reduced ability to exercise, genetic factors, or the owner feeding the dog more often than necessary [2, 34]. Our results showed that the body fat percentage in dogs tended to increase with age.

The combined prevalence of overweight or obesity in domestic canine populations has been reported to 41% [2]. The percentage of obese dogs was 34.1% in the USA [15], 25% in Australia [2], and 44.4% in China [35]. In addition, the rate of overweight dogs in the UK increased from 21% in 2006 to 35% in 2009, and it is estimated that approximately half of all dogs in the UK are currently overweight [36]. These results showed that the combined percentages of overweight and obese dogs in intact male, neutered male, intact female, and spayed female dogs are 17.64%, 27.63%, 17.33%, and 30.77%, respectively. Overall, the combined percentage of overweight and obese dogs in this study was 23.82%. This figure is lower than that in the reports from the aforementioned countries, probably due to the climate, housing conditions, and the feeding regime for the dogs. Furthermore, Thailand is a tropical country where the temperature is consistently > 35^o^C, and more than half of the dogs in this study stayed outdoors (data not shown). In addition, the quantity and quality of the dogs’ feed will depend on the dog owners’ status.

In this study, the percentages of overweight and obese dogs in the neutered male and spayed female categories were higher than those in the intact dogs, which were consistent with other reports that the obesity rate tended to increase with age and in neutered and spayed dogs [35]. Neutered or spayed dogs have lost body androgen or estrogen (sex hormones), which act on the central nervous system and stimulate roaming behavior and physiology activity; this loss decreases the metabolic rate, and their energy needs are lower than that in the normal dogs [37–39], and the average body fat in spayed female and neutered male dogs is approximately 4% more than that in intact male dogs [40]. However, in this study, the body fat percentages of spayed female and neutered male dogs did not significantly differ, which are in contrast with the findings of another study that neutering increases the risk of obesity in male dogs but not in female ones [41].

The food type did not affect the body fat percentage in this study; this was probably because the owner controlled the dogs’ diet, selected commercial pet food, or cooked using ingredients that were rich in nutrients and poor in calories [42–44]; although, a previous study found that the owners of dogs with high BCS have less perceived control over feeding and exercise [45]. It is a limitation of this study that data on the nutritional content of food were not collected. Neutered male dogs that had activity outside the house have a high appetite; however, their metabolism is low [38]. Therefore, the owners may increase the volume of food, which may have subsequently caused them to have higher body fat than those that never had activity outside the house. The body fat percentage of dogs those had snacks tended to increase and significantly higher in neutered male dogs, which may have been affected by the nutritional value of the snacks [46]. Neutered male dogs that had more than 1 meal/day had significantly lower body fat than dogs had one meal/day, which may have been affected by the volume of food per meal; this is consistent with the findings of a study that found that animals with normal weight had two portions by their owners, whereas obese animals were more often fed their meal in one or three-plus portions [47]. All dogs aged ≥ 5 years had significantly higher body fat percentage than those in the 1–2 years old group, probably because age is a composite factor for subcutaneous fat in dogs [48].

## Conclusion

The body fat percentage in dogs can be estimated by BIA, particularly the IBF-D02 model. It was determined to be a valid and effective measurement tool for predicting the body fat percentage in dogs. The linear model for predicting body fat percentages (y) by BCS (x), y = 0.84+8.36x, will be useful for clinicians and dog owners to estimate the body fat percentage in dogs and could be used as a prediction model for stratified medicine research and treatment strategies. The 9-point BCS scale, food intake, volume of energy utilization, and type of snack should be applied to a linear model to increase the accuracy of this model in future studies.

## Authors’ Contributions

TP: Designed the study. NN: Conducted the study. PA, TP, SC, and TJ: Interpreted the results and drafted the manuscript. TJ: Revised and finalized the manuscript. WR and JP: Supervised the bioelectric impedance analysis and clinical techniques. All authors have read and approved the final manuscript.
